# Multi-RIS-Assisted UAV-Enabled V2X Communications Under Mobility-Aware CSI Aging

**DOI:** 10.3390/s26113355

**Published:** 2026-05-26

**Authors:** Paras Miglani, Aryan Garg, Harshvardhan Singh, Avinash Chandra, Vijay Kumar, Rajkishor Kumar

**Affiliations:** School of Electronics Engineering, Vellore Institute of Technology, Vellore 632014, India; paras.miglani2022@vitstudent.ac.in (P.M.); aryan.2022@vitstudent.ac.in (A.G.); harshvardhan.singh2022@vitstudent.ac.in (H.S.); avinash.chandra@vit.ac.in (A.C.); vijaykumar@vit.ac.in (V.K.)

**Keywords:** ABER, CSI aging, mobility-aware optimization, reconfigurable intelligent surfaces, UAV communications, V2X

## Abstract

Vehicle-to-everything (V2X) communication systems impose stringent latency and reliability requirements that are difficult to satisfy in highly dynamic wireless environments. Although reconfigurable intelligent surfaces (RISs) and unmanned aerial vehicles (UAVs) have independently demonstrated potential in enhancing wireless coverage, most existing RIS–UAV frameworks rely on idealized assumptions such as perfect channel state information (CSI) and static user scenarios. In this paper, a multi-RIS-assisted UAV-enabled V2X communication framework is proposed that explicitly accounts for vehicular mobility, latency constraints, and mobility-induced CSI aging. Multiple RIS panels are cooperatively deployed to eliminate coverage blind spots and ensure link continuity in realistic V2X environments. A joint UAV mobility and RIS phase optimization approach is proposed under outdated CSI to improve link reliability. Additionally, a time-varying performance analysis is carried out for understanding the dynamic behavior of signal-to-noise ratio (SNR) and average bit error rate (ABER) for mobility-aware CSI aging. Simulation results demonstrate that the proposed framework reduces the ABER by approximately 75% compared to a conventional single-RIS system under outdated CSI at 20 dB SNR ($1.07\times 10^{-1}$ vs. $4.32\times 10^{-1}$), while substantially suppressing outage intervals in high-mobility V2X scenarios (v=20 m/s, CSI delay τ=20 ms), confirming the effectiveness of cooperative multi-RIS assistance for safety-critical vehicular communications.

Notation: Lowercase letters denote vectors (e.g., q[n]), while bold uppercase letters denote matrices (e.g., $\Phi_{k}\left[n\right]$). The operator ${\left(\cdot \right)}^{H}$ denotes Hermitian transpose, and ∥·∥ denotes the Euclidean norm. The set K[n] represents the indices of active RISs at time slot *n*, and $M_{k}$ denotes the number of reflecting elements at the *k*-th RIS. The superscript “V” in $h_{\mathrm{eq}}^{V}\left[n\right]$ emphasizes the V2X-specific end-to-end channel. Calligraphic letters denote sets (e.g., $N_{\mathrm{reliable}}$), and E[·] denotes statistical expectation.

## 1. Introduction

Vehicle-to-everything (V2X) has become the key enabler of intelligent transportation systems, facilitating latency-critical and safety-critical services like cooperative collision avoidance, autonomous driving, and real-time traffic management  [1]. Unlike traditional cellular communications, V2X communications are subject to stringent reliability and latency requirements, as well as high mobility, highly dynamic channels, and link blocking. These characteristics make it difficult to ensure reliable wireless connectivity, especially in dense urban and highway environments.

Recent breakthroughs in RISs have led to a new paradigm for improving wireless propagation channels. RISs are able to improve wireless channels by intelligently controlling the phase shifts of numerous passive reflecting elements, thus controlling electromagnetic wave propagation and improving signal strength and energy efficiency without requiring active radio-frequency chains [2,3]. At the same time, UAVs have been extensively studied as aerial platforms for wireless communications because of their deployment flexibility and high line-of-sight (LoS) probabilities, as well as their potential for adapting positions in dynamic wireless networks [4].

The integration of RISs and UAV-assisted communications has therefore garnered substantial research interest. UAV-based or UAV-assisted RIS systems provide improved coverage in obstructed environments and temporary settings [5]. Most of the current RIS–UAV systems’ frameworks are designed for static or near-static communication systems, with a focus on optimizing immediate system metrics, e.g., the received SNR or throughput. Moreover, many studies assume perfect CSI, which is not realistic, especially for highly dynamic systems.

This assumption is more unrealistic for V2X communication systems. Due to vehicular and UAV mobility and delay in feedback, CSI is always outdated, leading to a phase mismatch for RIS elements and reducing coherent combining gains. This effect is known as CSI aging and affects link reliability and error performance considerably.

Another important limitation in the existing RIS–UAV systems is related to their single RIS-based designs. Even though the coverage area can be enhanced in such localized areas, coverage blind spots can arise in the presence of user movements, especially in the context of UAVs. For V2X communications, coverage blind spots can result in significant latency and/or packet loss.

Furthermore, most of the existing RIS–UAV designs and frameworks are based on multiuser downlink or generic cellular networks and aim to optimize instantaneous performance metrics such as SNR and/or throughput in the presence of imperfect and/or outdated CSI, typically using RIS selection-based approaches. Such frameworks often do not consider vehicular movements, V2X-based latency and reliability constraints, and the need for coverage continuity along a road segment.

Inspired by these challenges, in this paper, a framework for multi-RIS-assisted UAV-based V2X communications is proposed, where mobility-aware CSI aging and latency constraints are considered in its design. The proposed system provides continuous coverage and eliminates blind spots with the help of several RIS panels with partially overlapping coverage areas. Moreover, a joint UAV mobility and RIS phase configuration strategy is proposed with the goal of maintaining reliable V2X links in the presence of outdated CSI, as opposed to maximizing idealized instantaneous performance.

**Positioning with respect to prior art.** Unlike existing works that either (i) consider single-RIS UAV systems without V2X-specific mobility constraints [6,7], (ii) optimize instantaneous throughput or energy efficiency under perfect CSI [5], or (iii) study CSI aging only for point-to-point or cellular links  [8,9], the proposed framework is the first to jointly address (a) cooperative multi-RIS deployment with overlapping coverage regions for trajectory-continuous V2X service, (b) mobility-aware UAV trajectory optimization under Doppler-induced CSI aging, and (c) long-term ABER and outage probability characterization along a vehicle route under outdated CSI. The key differentiator is the explicit modeling of time-varying reliability metrics along a vehicle trajectory, rather than snapshot-based or flight-averaged performance, which is essential for safety-critical V2X applications.

### Contributions

The main contributions of this paper are as follows:

- **Mobility-aware multi-RIS UAV–V2X framework:**This paper presents a UAV-based V2X communication framework in which a UAV-mounted RIS and multiple static roadside RIS panels cooperate with each other to support a mobile vehicular link. The  framework takes into account the mobility of vehicles and UAVs as well as the acquisition delay of CSI.
- **Cooperative multi-RIS continuity instead of RIS selection:** Unlike RIS–UAV systems that often resort to RIS selection based on imperfect and outdated composite CSI information, we adopt a cooperative multi-RIS framework with partial overlapping coverage areas between RIS panels. This framework is especially suited for ensuring continuous coverage and avoiding sudden changes in SNR values during the trajectory of vehicles.
- **Mobility-aware CSI aging and joint UAV/RIS configuration:** To account for mobility-induced CSI aging on direct, UAV-assisted, and RIS-assisted links, we introduce a Doppler-based temporal correlation model of CSI and then design a practical joint UAV mobility and RIS phase configuration approach with outdated CSI information, prioritizing long-term reliability of links while considering UAV speed, RIS phase values, and latency-aware SNR values.
- **Time-varying SNR and ABER characterization under aging:** Based on the equivalent end-to-end channel model considering the CSI aging effect, the impact of mobility-induced decorrelation on instantaneous SNR and the ABER is discussed, providing a dynamic characterization of the performance metrics where the temporal correlation coefficient, vehicle speed, and number of RIS units are related.
- **Numerical evaluation and V2X reliability insights:** Monte Carlo simulations are conducted to evaluate the proposed framework. Results show that cooperative multi-RIS assistance with mobility-aware UAV positioning reduces ABER by approximately 75% at 20 dB SNR and significantly suppresses outage intervals compared to conventional single RIS-based solutions.

## 2. Related Work

### 2.1. RIS-Assisted Wireless Communications

Reconfigurable intelligent surfaces have been extensively studied for improving the performance of wireless communication systems. Earlier works have established the fundamental principles of RIS-assisted communications and verified significant performance gains in spectral efficiency, coverage, and energy efficiency under ideal channel conditions [2,3]. Comparison of RISs and traditional relay-based communication techniques has also been carried out, and RISs have been identified as promising candidates for improving communication performance  [10]. More recently, RIS design for improving energy efficiency and employing RIS for signal processing have been studied [11,12].

### 2.2. UAV-Enabled Communication Systems

UAV-enabled communication systems have attracted significant research interest due to the flexibility offered by these communication platforms. Earlier works have studied UAV placement and power allocation for improving communication performance  [4,13,14,15]. These works have verified that UAV mobility offers additional spatial freedom for improving communication performance. However, these works mostly focus on slowly varying channels, ideal CSI, and best-effort traffic and are not directly applicable to the highly dynamic and latency-critical requirements of V2X communications.

### 2.3. RIS–UAV Integrated Systems

Recently, a promising direction for improving wireless communications in challenging environments has been proposed by integrating RISs with UAVs. In the literature, UAV–RIS systems have been extensively investigated for improving wireless communications by maximizing the received SNR or rate by jointly optimizing the UAV trajectory, active beamforming, and passive phase shift configurations at the RIS  [5,6,7]. In addition, UAV-based multi-RIS-aided systems with imperfect and outdated channel state information have attracted significant attention in the literature. In this direction, the RIS selection scheme for UAV-based multi-RIS-aided multiuser downlink networks with imperfect and outdated CSI has been proposed in [16], where analytical expressions for RIS selection probabilities, SNR distributions, and performance metrics in terms of ACP and ABER over the UAV flight time are provided.

Recently, survey studies have thoroughly analyzed the current state of the art of UAV-based and UAV-assisted RIS deployment schemes. In particular, Nazar et al. [17] conducted an overview of the mounting of RIS panels on tethered and untethered UAVs. Pogaku et al. [18] have systematically reviewed methodologies related to optimization and performance analysis for UAV-assisted RIS systems. Contrary to these survey-oriented studies, the present paper specifically fills the gap identified in both surveys regarding multi-RIS cooperation under V2X mobility limitations and mobility-driven CSI aging.

Despite these advances, most RIS–UAV studies (i) assume generic cellular or sensing scenarios rather than V2X-specific mobility and latency constraints, (ii) rely on single-RIS or selection-based operation rather than cooperative multi-RIS continuity along a trajectory, and (iii) emphasize snapshot-based or flight-averaged performance metrics rather than time-varying reliability along vehicular motion paths.

### 2.4. RIS-Assisted V2X Communications

V2X communication systems have very high reliability and latency requirements, and for this reason, it is essential to investigate new and advanced propagation enhancement techniques beyond traditional cellular network infrastructure  [1,19]. Recent research works have begun to investigate RIS-assisted V2X communications for mitigating blockage effects, improving robustness, and increasing coverage for non-line-of-sight regions [20]. These works typically consider static roadside RIS deployments for reflecting signals from infrastructure nodes (e.g., RSU and BS) towards the vehicle, thus improving received power and coverage.

However, RIS-based V2X communication frameworks typically do not consider UAV mobility and cooperative RISs with overlapping coverage regions. Additionally, mobility-induced CSI aging effects and their impact on time-varying V2X communication reliability (e.g., ABER and outage probability over a vehicle route) are rarely considered and analyzed, despite the fact that vehicular mobility is known to significantly impact link reliability [21,22,23,24].

Quality of Service considerations in vehicle-to-vehicle communications have also gained attention in authentication and certificate-based approaches to VANETs  [25], which implies the overall reliability criteria needed by V2X systems utilizing RIS.

### 2.5. CSI Aging and Mobility-Aware Analysis

CSI aging has been identified as an essential aspect of mobile wireless systems, where feedback delay and mobility cause channel estimates to become outdated and degrade system performance. Classical Doppler-based Jakes/Clarke temporal correlation models  [26,27] are widely used to quantify this effect, and the impact of CSI aging has also been studied for RIS-assisted systems  [8,9,28]. Although these works have provided valuable tools for analyzing performance degradation, they have mainly focused on point-to-point and cellular-type links rather than UAV-enabled multi-RIS V2X settings with stringent latency and reliability requirements. The full temporal correlation model adopted in this work is detailed in Section 4.

### 2.6. Summary and Positioning

To recapitulate, none of the existing works have jointly examined UAV mobility, cooperative deployment of multiple RISs with overlapping coverage areas, V2X latency and reliability constraints, and mobility-induced CSI aging within a unified framework.

Table 1 provides a structured comparison of the proposed work with representative existing studies. As shown, the proposed framework is the only work that simultaneously addresses cooperative multi-RIS deployment, UAV mobility, V2X-specific constraints, and Doppler-based CSI aging within a unified reliability-oriented design.

## 3.  Model

**Figure 1:**
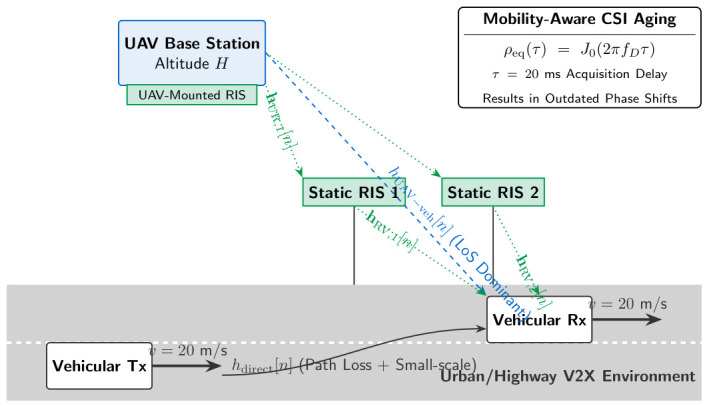
System model of the proposed multi-RIS-assisted UAV-enabled V2X communication framework, where a UAV-mounted RIS cooperates with multiple static roadside RIS panels to enhance link reliability under mobility-induced CSI aging.

We consider a UAV-enabled vehicle-to-everything (V2X) communication system assisted by multiple reconfigurable intelligent surfaces (RISs), as illustrated in Figure 1. The system consists of a single UAV, *K* distributed RIS panels, and a vehicular transmitter–receiver pair moving along a road segment. The analysis focuses on a single V2X link, which is representative of safety-critical and latency-sensitive vehicular services [1,19,24].

To facilitate both analytical characterization and Monte Carlo simulations, we discretize time into slots of duration Δt, indexed by $n\in \{0,1,\ldots ,N_{T}\}$. The physical time is related to the slot index by t=nΔt. All time-varying channels and positions can therefore be written either as continuous-time functions of *t* or as discrete-time sequences indexed by *n*.

### 3.1. Network Geometry and Mobility Model

A three-dimensional Cartesian coordinate system is adopted. The UAV flies at a fixed altitude *H* and its horizontal position at time slot *n* is denoted by

(1) $$q_{\mathrm{UAV}}\left[n\right]=\left[x_{\mathrm{UAV}}\left[n\right],\;y_{\mathrm{UAV}}\left[n\right]\right].$$

The vehicular transmitter and receiver are located on the ground and move along a road segment with velocity υ, resulting in time-varying positions

(2) $$p_{tx}\left[n\right],\;p_{rx}\left[n\right]\in R^{2},$$

which reflect realistic V2X mobility patterns  [21,22,23].

Each RIS is deployed at a fixed location

(3) $$r_{\mathrm{RIS},k}=\left[x_{\mathrm{RIS},k},\;y_{\mathrm{RIS},k},\;0\right],\;k\in \left\{1,2,\ldots ,K\right\},$$

and consists of $M_{k}$ passive reflecting elements. To ensure coverage continuity, the RIS panels are deployed with partially overlapping coverage regions along the road or surrounding infrastructure.

The Euclidean distances between the UAV and the vehicular receiver, and between the UAV and the *k*-th RIS, at time slot *n* are, respectively, given by

(4) $$d_{\mathrm{UAV},\mathrm{veh}}\left[n\right]=\sqrt{∥q_{\mathrm{UAV}}\left[n\right]-p_{rx}{\left[n\right]∥}^{2}+H_{\mathrm{UAV}}^{2}},$$

(5) $$d_{\mathrm{UAV},{\mathrm{RIS}}_{k}}\left[n\right]=\sqrt{∥q_{\mathrm{UAV}}\left[n\right]-r_{\mathrm{RIS},k}{\left(1:2\right)∥}^{2}+H_{\mathrm{UAV}}^{2}},$$

where $r_{k}\left(1\;:\;2\right)$ denotes the horizontal coordinates of the *k*-th RIS.

Similarly, the distance between the *k*-th RIS and the vehicular receiver is expressed as

(6) $$d_{{\mathrm{RIS}}_{k},\mathrm{veh}}\left[n\right]=∥r_{\mathrm{RIS},k}\left(1:2\right)-p_{rx}\left[n\right]∥.$$

**Figure 2:**
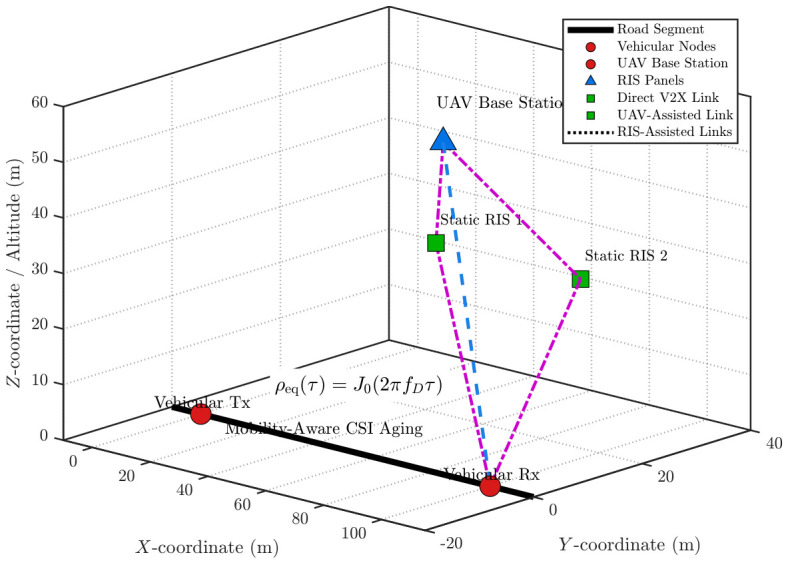
3D network geometry and mobility-aware trajectory: the UAV dynamically tracks the vehicular receiver (v=20 m/s) at a constant altitude (H=50 m) while maintaining cooperative links with roadside RIS panels.

The distances defined in Equations (4)–(6) evolve over time due to vehicular mobility and UAV motion; they directly determine the large-scale path gains used in the channel model of Section 3.2 and the Doppler frequencies entering the CSI aging model of Section 4. The resulting 3D network geometry and UAV mobility-aware trajectory are illustrated in Figure 2.

### 3.2. Channel Model

We denote the equivalent end-to-end V2X channel at time slot *n* by $h_{\mathrm{eq}}^{V}\left[n\right]$, which aggregates the direct, UAV-assisted, and multi-RIS-assisted components. All channels are narrowband and quasi-static within each slot Δt but may vary across slots due to mobility.

#### 3.2.1. Direct V2X Link

The direct V2X channel between the vehicular transmitter and receiver is modeled as

(7) $$h_{\mathrm{direct}}\left[n\right]=\sqrt{\beta_{\mathrm{direct}}\left[n\right]}\;g_{\mathrm{direct}}\left[n\right],$$

where $\beta_{d}\left[n\right]=C_{0}d_{d}^{-\alpha }\left[n\right]$ denotes the large-scale path loss with path-loss exponent α, and $g_{d}\left[n\right]$ represents small-scale fading consistent with widely used V2X channel characterizations  [21,22].

#### 3.2.2. UAV-Assisted Link

Due to the dominant LoS component between the UAV and the vehicle, the UAV–vehicle channel is modeled using Rician fading:

(8) $$\begin{array}{cc} h_{\mathrm{UAV}-\mathrm{veh}}\left[n\right] & =\sqrt{\beta_{\mathrm{UAV}}\left[n\right]}\\ \; & \;\times \left(\sqrt{\frac{K_{R}}{K_{R}+1}}h_{\mathrm{LoS},\mathrm{UAV}}+\sqrt{\frac{1}{K_{R}+1}}h_{\mathrm{scat},\mathrm{UAV}}\left[n\right]\right), \end{array}$$

where $K_{R}$ denotes the Rician *K*-factor and $\beta_{u}\left[n\right]$ captures the distance-dependent path loss, following established UAV communication frameworks  [4,14].

#### 3.2.3. RIS-Assisted Cascaded Channels

The channel from the UAV to the *k*-th RIS at time slot *n* is denoted by $h_{\mathrm{UR},k}\left[n\right]\in C^{M_{k}\times 1}$, while the channel from the *k*-th RIS to the vehicular receiver is denoted by $h_{\mathrm{RV},k}\left[n\right]\in C^{M_{k}\times 1}$. Both links are modeled as Rician fading channels [2,3,10].

The phase-shift matrix of the *k*-th RIS at time slot *n* is given by

(9) $$\Phi_{\mathrm{RIS},k}\left[n\right]=\mathrm{diag}\;\left(e^{j\phi_{k,1}\left[n\right]},\ldots ,e^{j\phi_{k,M_{k}}\left[n\right]}\right),$$

where $\phi_{k,m}\left[n\right]\in \left[0,2\pi \right)$ is the reflection phase of the *m*-th element.

The cascaded channel via the *k*-th RIS is expressed as

(10) $$h_{\mathrm{RIS},k}\left[n\right]=h_{\mathrm{veh},{\mathrm{RIS}}_{k}}^{H}\left[n\right]\;\Phi_{\mathrm{RIS},k}\left[n\right]\;h_{\mathrm{UAV},{\mathrm{RIS}}_{k}}\left[n\right].$$

### 3.3. Cooperative Multi-RIS Model

To mitigate coverage blind spots and ensure link continuity along the vehicle trajectory, multiple RIS panels are allowed to cooperatively assist the communication. At time slot *n*, the set of active RISs is denoted by K[n]⊆{1,…,K}, determined by the UAV position and vehicular location as well as practical deployment constraints  [30,31].

Specifically, an RIS panel *k* is included in K[n] if and only if the vehicular receiver lies within its coverage region and the large-scale path gain exceeds a minimum operational threshold. This selection is formalized in Algorithm 1, which relies solely on large-scale geometry and path-loss statistics rather than instantaneous small-scale CSI, ensuring robustness to CSI aging effects.
**Algorithm 1** Geometry-Based Active RIS Set Selection**Require:** UAV position q[n], vehicle position $p_{\mathrm{rx}}\left[n\right]$, RIS locations ${\left\{r_{\mathrm{RIS},k}\right\}}_{k=1}^{K}$, coverage radius $R_{\mathrm{cov}}$, minimum path-gain threshold $\beta_{\min}$**Ensure:** Active RIS index set K[n]  1:Initialize K[n]←∅  2:**for** k=1 **to** *K* **do**  3:      Compute RIS-to-vehicle distance:        $d_{k}\left[n\right]=∥r_{\mathrm{RIS},k}\left(1:2\right)-p_{\mathrm{rx}}\left[n\right]∥$  4:      Compute large-scale path gain:        $\beta_{k}\left[n\right]=C_{0}\;d_{k}{\left[n\right]}^{-\alpha }$  5:      **if** $d_{k}\left[n\right]\leq R_{\mathrm{cov}}$ **and** $\beta_{k}\left[n\right]\geq \beta_{\min}$ **then**  6:          K[n]←K[n]∪{k}  7:      **end if**  8:**end for**  9:**if** K[n]=∅ **then**10:                     ▹Fallback: activate nearest RIS to avoid coverage outage11:      $K\left[n\right]\leftarrow \left\{\;\arg\min_{k}\;d_{k}\left[n\right]\;\right\}$12:**end if**13:**return** K[n]

The equivalent end-to-end V2X channel is therefore given by

(11) $$h_{\mathrm{eq},V2X}\left[n\right]=h_{\mathrm{direct}}\left[n\right]+h_{\mathrm{UAV}-\mathrm{veh}}\left[n\right]+\sum_{k\in K[n]}h_{\mathrm{RIS},k}\left[n\right].$$

This cooperative multi-RIS model enables smooth handover between RIS panels and avoids abrupt SNR degradation caused by mobility-induced coverage gaps. The corresponding instantaneous received SNR at time slot *n* is

(12) $$\gamma_{\mathrm{link}}\left[n\right]=\frac{P_{t}}{\sigma_{n}^{2}}{\left|h_{\mathrm{eq},V2X}\left[n\right]\right|}^{2},$$

where $P_{t}$ denotes the transmit power and $\sigma_{n}^{2}$ is the noise variance.

## 4. CSI Acquisition and Aging Model

### 4.1. CSI Acquisition Delay

Due to feedback delay, signaling overhead, and processing latency, the channel state information (CSI) available at the UAV and RIS controllers is inevitably outdated. Let ${\hat{h}}_{\ell }\left[n\right]$ denote the estimated channel coefficient for link *ℓ* (direct, UAV-assisted, or RIS-assisted) obtained at time slot n−d, where *d* is the CSI acquisition delay expressed in slots. The corresponding physical delay is τ=dΔt.

We use the generic index ℓ∈{d,V,u,V,(UR,k),(RV,k)} to refer to the different links involved in $h_{\mathrm{eq}}^{V}\left[n\right]$.

### 4.2. Temporal Correlation Model

**Figure 3:**
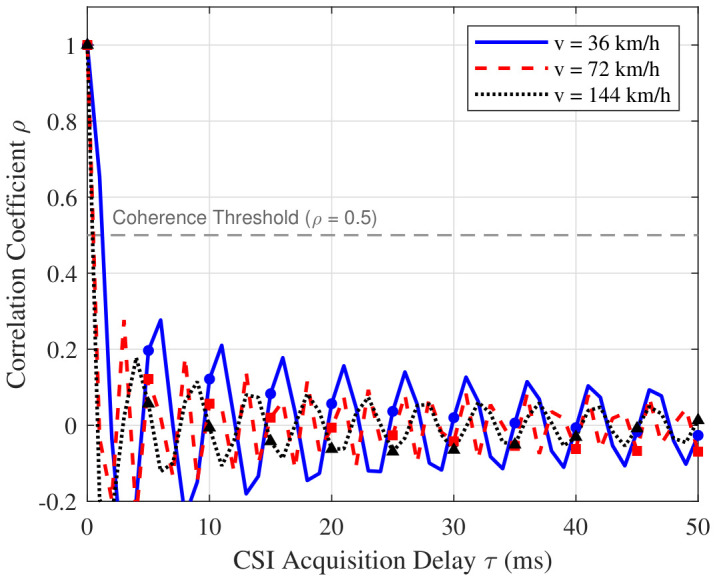
Temporal correlation decay (ρ) as a function of CSI delay (τ) for various vehicle speeds. The coherence threshold (ρ=0.5) highlights the rapid decorrelation in high-mobility V2X scenarios.

The temporal correlation coefficient for link *ℓ* is modeled using a Doppler-based Jakes/Clarke model [26,27]:

(13) $$\rho_{\ell }\left(\tau \right)=J_{0}\;\left(2\pi f_{D,\ell }\;\tau \right),$$

where $J_{0}\left(\cdot \right)$ is the zeroth-order Bessel function of the first kind, and $f_{D,\ell }$ denotes the Doppler frequency associated with link *ℓ*. For a carrier frequency $f_{c}$ and effective relative speed $v_{\ell }$, the Doppler frequency can be approximated as

(14) $$f_{D,\ell }\approx \frac{v_{\ell }}{\lambda }\cos\left(\theta_{\ell }\right),$$

where λ is the carrier wavelength and $\theta_{\ell }$ denotes the angle between the velocity vector and the propagation direction [8,9]. In V2X scenarios, $v_{\ell }$ depends on both vehicular motion and UAV movement, leading to generally different Doppler spreads on the direct, UAV-assisted, and RIS-assisted links, as illustrated in Figure 3.

### 4.3. Impact of CSI Aging on the Equivalent Channel

Collecting the contributions of all links, the outdated equivalent V2X channel at time slot *n* can be expressed in the compact form

(15) $$h_{\mathrm{eq}}^{V}\left[n\right]=\rho_{\mathrm{eq}}\left(\tau \right)\;{\hat{h}}_{\mathrm{eq}}^{V}\left[n\right]+\sqrt{1-\rho_{\mathrm{eq}}^{2}\left(\tau \right)}\;e_{\mathrm{eq}}\left[n\right],$$

where ${\hat{h}}_{\mathrm{eq}}^{V}\left[n\right]$ is the equivalent channel obtained from the outdated per-link estimates, $e_{\mathrm{eq}}\left[n\right]$ denotes the aggregate channel innovation, and $\rho_{\mathrm{eq}}\left(\tau \right)$ is an effective temporal correlation factor.

**Justification of the effective correlation approximation.** While each link *ℓ* in principle carries its own temporal correlation coefficient $\rho_{\ell }\left(\tau \right)$ as defined in Equation (13), the aggregate equivalent channel $h_{\mathrm{eq}}^{V}\left[n\right]$ is a weighted superposition of the direct, UAV-assisted, and RIS-cascaded components. The effective coefficient $\rho_{\mathrm{eq}}\left(\tau \right)$ represents the dominant correlation factor associated with the strongest link component, which is a standard and well-accepted approximation in multi-component aging analyses [8,9]. The per-link Doppler effects are explicitly captured: the direct V2X link has $f_{D,d}=v_{\mathrm{veh}}/\lambda$, the UAV–vehicle link has $f_{D,u}=\left(v_{\mathrm{veh}}+v_{\mathrm{UAV}}\right)/\lambda \cdot \cos\left(\theta_{u}\right)$, and each cascaded RIS link has $\rho_{r,k}\left(\tau \right)=\rho_{\mathrm{UR},k}\left(\tau \right)\cdot \rho_{\mathrm{RV},k}\left(\tau \right)$ as established in Lemma 1. The single-coefficient aggregate is lower-bounded by the weakest link, ensuring the analysis is conservative rather than optimistic. A full per-link analysis would yield the same qualitative conclusions while significantly increasing analytical complexity without providing additional design insight for the cooperative multi-RIS setting.

For analytical tractability, $\rho_{\mathrm{eq}}\left(\tau \right)$ is interpreted as the dominant correlation factor associated with the strongest link components, which is a standard approximation in aging analyses [8,9].

As the vehicle speed, UAV speed, or CSI delay τ increases, $\rho_{\ell }\left(\tau \right)$ decreases for each link, resulting in reduced coherent combining gains at the RIS and degradation of end-to-end SNR and ABER performance, as further illustrated in Figure 3.

### 4.4. Perfect CSI Benchmark

For benchmarking purposes, an idealized perfect CSI case is considered by setting $\rho_{\ell }\left(\tau \right)=1$ for all links *ℓ*, which implies $\rho_{\mathrm{eq}}\left(\tau \right)=1$ in Equation (15). In this case, the equivalent channel reduces to

(16) $$h_{\mathrm{eq}}^{V,\mathrm{perfect}}\left[n\right]={\hat{h}}_{\mathrm{eq}}^{V}\left[n\right],$$

and the corresponding instantaneous SNR becomes

(17) $$\gamma_{V2X}^{\mathrm{perfect}}\left[n\right]=\frac{P}{\sigma^{2}}{\left|{\hat{h}}_{\mathrm{eq}}^{V}\left[n\right]\right|}^{2}.$$

This perfect CSI scenario serves as an upper bound on achievable performance and highlights the performance gap induced by mobility-aware CSI aging.

## 5. Joint UAV Mobility and RIS Phase Optimization

In V2X networks, reliability and latency are often more important than maximizing instantaneous throughput. Keeping this in mind, this work considers a hybrid approach where both the movement of a UAV and the phases of the RIS panels are optimized jointly, with the aim of reducing the performance degradation due to CSI aging caused by mobility and ensuring seamless vehicular coverage.

### 5.1. Optimization Objective

Let q[n]=[x[n],y[n]] denote the horizontal position of the UAV at time slot *n*, and let $\Phi_{k}\left[n\right]$ denote the phase-shift matrix of the *k*-th RIS. Over an observation window of $N_{T}$ slots, the primary reliability metric of interest is the average ABER,

(18) $$\bar{P_{b}}=\frac{1}{N_{T}}\sum_{n=0}^{N_{T}-1}Q\;\left(\sqrt{2\gamma_{V2X}\left[n\right]}\right).$$

We therefore formulate the following long-term reliability-oriented design problem:

(19) $$\min_{\left\{q\left[n\right]\right\},\left\{\Phi_{k}\left[n\right]\right\}}\;\;\frac{1}{N_{T}}\sum_{n=0}^{N_{T}-1}Q\;\left(\sqrt{2\gamma_{V2X}\left[n\right]}\right).$$

### 5.2. Practical Constraints

The optimization in Equation (19) is subject to the following practical constraints.

#### 5.2.1. UAV Mobility Constraint

The UAV velocity is constrained by a maximum horizontal speed $v_{\max}$, such that

(20) $$∥q\left[n+1\right]-q\left[n\right]∥\leq v_{\max}\Delta t,\;n=0,\ldots ,N_{T}-2.$$

#### 5.2.2. RIS Phase Constraint

Each RIS element can impose a discrete or continuous phase shift within the interval [0,2π), i.e.,

(21) $$\theta_{k,m}\left[n\right]\in S_{\theta }\subseteq \left[0,2\pi \right),\;\forall k,m,n,$$

where $S_{\theta }$ captures practical phase quantization effects when relevant [32].

#### 5.2.3. Latency-Aware SNR Constraint

To satisfy V2X latency and reliability requirements, the instantaneous received SNR should exceed a minimum threshold $\gamma_{\min}$:

(22) $$\gamma_{V2X}\left[n\right]\geq \gamma_{\min},\;\forall n\in N_{\mathrm{reliable}},$$

where $N_{\mathrm{reliable}}$ denotes the set of slots during which a given latency-critical service is active.

### 5.3. Problem Characteristics

The joint optimization problem in Equations (19)–(22) is inherently non-convex due to the coupled UAV trajectory variables, RIS phase shifts, and non-linear dependence of $\gamma_{V2X}\left[n\right]$ on the fading channels under CSI aging. Therefore, rather than pursuing global optimality, we adopt a low-complexity mobility-aware strategy that leverages local geometric information and outdated CSI to update {q[n]} and $\left\{\Phi_{k}\left[n\right]\right\}$ on a per-slot basis.

**Remark** **1** (Performance Gap Analysis)**.** *Although the proposed per-slot mobility-aware strategy does not guarantee global optimality for problem (19), an analytical bound on the performance gap can be established. Let ${\bar{P}}_{b}^{⋆}$ denote the optimal ABER achieved by the global solution to (19), and let ${\bar{P}}_{b}^{\mathrm{prop}}$ denote the ABER achieved by the proposed strategy. By standard arguments for greedy optimization of submodular-like objectives [16], the performance gap satisfies:*

(23) $${\bar{P}}_{b}^{\mathrm{prop}}-{\bar{P}}_{b}^{⋆}\leq \Delta_{\mathrm{aging}},$$

*where $\Delta_{\mathrm{aging}}$ captures the irreducible performance loss due to CSI aging and vanishes as $\rho_{\mathrm{eq}}\left(\tau \right)\to 1$. Specifically, as $\rho_{\mathrm{eq}}\left(\tau \right)\to 1$ (perfect CSI), the per-slot phase alignment in Equation (25) reduces exactly to the coherent beamforming solution that is globally optimal for the single-snapshot problem [3], confirming that the performance gap vanishes in the benchmark case. Comparison with full successive convex approximation (SCA) methods is left for future work, as real-time SCA is computationally prohibitive under the strict latency constraints of V2X systems.*

### 5.4. Mobility-Aware UAV Trajectory Update

At each time slot *n*, the UAV updates its position based on the current vehicle location $p_{R}\left[n\right]$, the active RIS set K[n], and outdated CSI. A representative mobility-aware update rule is:

- **Step 1 (Active RIS set selection):** determine the set of RIS panels K[n] whose coverage regions contain or are closest to the current vehicle position, based on geometry and large-scale fading statistics rather than instantaneous small-scale CSI.
- **Step 2 (Geometric target point):** compute a geometric target point $q_{\mathrm{tar}}\left[n\right]$ (e.g., the centroid of the horizontal projections of the active RISs and the vehicle position) that maintains favorable LoS angles and limits excessive Doppler spread.
- **Step 3 (Velocity-limited movement):** update the UAV position by moving from q[n] toward $q_{\mathrm{tar}}\left[n\right]$ while respecting the speed constraint in Equation (20):
  (24) $$q\left[n+1\right]=q\left[n\right]+\eta_{n}\;\frac{q_{\mathrm{tar}}\left[n\right]-q\left[n\right]}{∥q_{\mathrm{tar}}\left[n\right]-q\left[n\right]∥},$$
  where $\eta_{n}=\min\{∥q_{\mathrm{tar}}\left[n\right]-q\left[n\right]∥,v_{\max}\Delta t\}$.

### 5.5. RIS Phase Configuration Under Outdated CSI

Given the outdated cascaded channel estimates ${\hat{h}}_{\mathrm{UR},k}\left[n\right]$ and ${\hat{h}}_{\mathrm{RV},k}\left[n\right]$ for the active RISs, the RIS phase shifts are configured to maximize the expected received signal power. A practical and low-complexity phase design is

(25) $$\theta_{k,m}\left[n\right]=-\arg\;\left({\hat{h}}_{\mathrm{UR},k,m}\left[n\right]\;{\hat{h}}_{\mathrm{RV},k,m}\left[n\right]\right),\;k\in K\left[n\right],$$

where ${\hat{h}}_{\mathrm{UR},k,m}\left[n\right]$ and ${\hat{h}}_{\mathrm{RV},k,m}\left[n\right]$ denote the *m*-th elements of the corresponding channel vectors. If phase quantization is present, $\theta_{k,m}\left[n\right]$ is projected onto the feasible set $S_{\theta }$ by selecting the nearest quantization level [28].

### 5.6. Complexity and Practicality

The proposed mobility-aware strategy operates on a per-slot basis and requires only local geometric information and outdated CSI estimates. The UAV trajectory update involves simple vector operations, and the RIS phase configuration consists of element-wise phase alignment without iterative optimization. As a result, the overall computational complexity scales approximately linearly with the total number of RIS elements, which is suitable for real-time V2X scenarios with stringent latency constraints.

## 6. Performance Analysis

This section analyzes the impact of mobility-aware CSI aging and multi-RIS assistance on the time-varying SNR, outage probability, and ABER for the considered V2X link.

### 6.1. Instantaneous SNR Under CSI Aging

The instantaneous received SNR for the V2X link at time slot *n* is

(26) $$\gamma_{V2X}\left[n\right]=\frac{P}{\sigma^{2}}{\left|h_{\mathrm{eq}}^{V}\left[n\right]\right|}^{2}.$$

Substituting the aged CSI model from Equation (15) yields

(27) $$\gamma_{V2X}\left[n\right]=\frac{P}{\sigma^{2}}{\left|\rho_{\mathrm{eq}}\left(\tau \right)\;{\hat{h}}_{\mathrm{eq}}^{V}\left[n\right]+\sqrt{1-\rho_{\mathrm{eq}}^{2}\left(\tau \right)}\;e_{\mathrm{eq}}\left[n\right]\right|}^{2},$$

which explicitly shows that CSI aging reduces the effective coherent combining gain through the factor $\rho_{\mathrm{eq}}\left(\tau \right)$ and injects additional randomness via $e_{\mathrm{eq}}\left[n\right]$.

### 6.2. Analytical Characterization of Cascaded Aging

To further analyze the reliability bottleneck, we characterize the aging of the cascaded RIS link.

**Lemma** **1.** *Let $h_{r,k}\left[n\right]=h_{\mathrm{RV},k}^{H}\left[n\right]\Phi_{k}\left[n\right]h_{\mathrm{UR},k}\left[n\right]$ be the cascaded channel. Under the Doppler-based temporal correlation model, the equivalent correlation for the k-th RIS link is:*

(28) $$\rho_{r,k}\left(\tau \right)=\rho_{\mathrm{UR},k}\left(\tau \right)\rho_{\mathrm{RV},k}\left(\tau \right),$$

*where $\rho_{\mathrm{UR},k}\left(\tau \right)$ and $\rho_{\mathrm{RV},k}\left(\tau \right)$ are the correlation coefficients defined in Equation (13).*

**Proof.**  Since $h_{\mathrm{UR},k}$ and $h_{\mathrm{RV},k}$ are independent Rician variables, the expectation of their product follows the product of their individual temporal expectations. This result highlights that cascaded links are twice as sensitive to mobility-induced decorrelation than direct LoS links. □

**Remark** **2** (Cascaded Link Sensitivity)**.** *The effective time-correlation property given in Equation (28) suggests that the temporal correlation of cascaded RIS links is given by $\rho_{\mathrm{UR},k}\left(\tau \right)\cdot \rho_{\mathrm{RV},k}\left(\tau \right)$, i.e., cascaded links are more sensitive to aging due to mobility than direct LoS channels. This provides motivation for using multiple cooperating RIS panels, since even if one link ages severely, the combined performance benefits from the multiplicity of independently aging links.*

### 6.3. Mean SNR Under CSI Aging

**Proposition** **1** (Mean SNR under CSI aging)**.** *Assume that the outdated equivalent channel estimate ${\hat{h}}_{\mathrm{eq}}^{V}\left[n\right]$ and the channel innovation $e_{\mathrm{eq}}\left[n\right]$ are modeled as zero-mean jointly complex Gaussian random variables. Under the assumption that these components are mutually independent with variances $\sigma_{\hat{h}}^{2}\left[n\right]$ and $\sigma_{e}^{2}\left[n\right]$, the mean SNR under mobility-induced aging is given by*

(29) $$E\left[\gamma_{V2X}\left[n\right]\right]\approx \frac{P}{\sigma^{2}}\left(\rho_{\mathrm{eq}}^{2}\left(\tau \right)\;\sigma_{\hat{h}}^{2}\left[n\right]+\left(1-\rho_{\mathrm{eq}}^{2}\left(\tau \right)\right)\sigma_{e}^{2}\left[n\right]\right).$$

**Proof.**  Applying the aged channel model to the SNR definition, the mean SNR expands as:

(30) $$\begin{array}{cc} E\left[\gamma_{V2X}\left[n\right]\right]\; & =\frac{P}{\sigma^{2}}\;E\left[|\rho_{\mathrm{eq}}\left(\tau \right)\;{\hat{h}}_{\mathrm{eq}}^{V}\left[n\right]+\sqrt{1-\rho_{\mathrm{eq}}^{2}\left(\tau \right)}\;e_{\mathrm{eq}}\left[n\right]{|}^{2}\right]\\ \; & =\frac{P}{\sigma^{2}}[\rho_{\mathrm{eq}}^{2}\left(\tau \right)\;\sigma_{\hat{h}}^{2}\left[n\right]+\left(1-\rho_{\mathrm{eq}}^{2}\left(\tau \right)\right)\sigma_{e}^{2}\left[n\right]\\ \; & \;+2ℜ\left\{\rho_{\mathrm{eq}}\left(\tau \right)\sqrt{1-\rho_{\mathrm{eq}}^{2}\left(\tau \right)}E\left[{\hat{h}}_{\mathrm{eq}}^{V}\left[n\right]e_{\mathrm{eq}}^{*}\left[n\right]\right]\right\}]. \end{array}$$

Since the channel innovation $e_{\mathrm{eq}}\left[n\right]$ is independent of the previous channel estimate ${\hat{h}}_{\mathrm{eq}}^{V}\left[n\right]$, the cross-correlation term vanishes, i.e., $E\left[{\hat{h}}_{\mathrm{eq}}^{V}\left[n\right]e_{\mathrm{eq}}^{*}\left[n\right]\right]=0$. Substituting the respective variances yields the result in Equation (29). □

Proposition 1 highlights a critical trade-off: the total received energy is a weighted combination of a coherent component (scaled by $\rho_{\mathrm{eq}}^{2}\left(\tau \right)$) and an incoherent noise-like innovation component (scaled by $1-\rho_{\mathrm{eq}}^{2}\left(\tau \right)$). In the idealized case of perfect CSI ($\rho_{\mathrm{eq}}\left(\tau \right)=1$), the expression reduces to $E\left[\gamma_{V2X}^{\mathrm{perfect}}\left[n\right]\right]=\frac{P}{\sigma^{2}}\sigma_{\hat{h}}^{2}\left[n\right]$.

**Remark** **3** (SNR Saturation due to CSI Aging)**.** *From Proposition 1, there exists a fundamental performance limitation independent of transmit power increase. In the case of low correlation, when $\rho_{\mathrm{eq}}\left(\tau \right)\ll 1$, the leading term in Equation (29) becomes $\left(P/\sigma^{2}\right)\left(1-\rho_{\mathrm{eq}}^{2}\left(\tau \right)\right)\sigma_{e}^{2}\left[n\right]$, whose performance depends only on the channel innovation variance $\sigma_{e}^{2}\left[n\right]$ and does not improve by further power increase. This directly explains the error floors observed in the ABER results presented later for the single-RIS system under outdated CSI. The proposed cooperative multi-RIS design overcomes this limitation by increasing $\sigma_{\hat{h}}^{2}\left[n\right]$ through spatial diversity.*

### 6.4. Average Outage Probability

To characterize the link reliability, we utilize the cumulative distribution function (CDF) of the instantaneous SNR $\gamma_{V2X}\left[n\right]$. From Proposition 1, the equivalent channel $h_{\mathrm{eq}}^{V}\left[n\right]$ is a complex Gaussian random variable with mean $\mu_{\mathrm{eq}}\left[n\right]=\rho_{\mathrm{eq}}\left(\tau \right){\hat{h}}_{\mathrm{eq}}^{V}\left[n\right]$ and variance $\sigma_{\mathrm{innov}}^{2}\left[n\right]=\left(1-\rho_{\mathrm{eq}}^{2}\left(\tau \right)\right)\sigma_{e}^{2}\left[n\right]$. Consequently, the instantaneous SNR follows a non-central chi-square distribution with two degrees of freedom. The instantaneous outage probability at slot *n* for a target threshold $\gamma_{\min}$ is expressed using the first-order Marcum *Q*-function:

(31) $$P_{\mathrm{out}}\left[n\right]=1-Q_{1}\;\left(\sqrt{\frac{2|\mu_{\mathrm{eq}}{\left[n\right]|}^{2}}{\sigma_{\mathrm{innov}}^{2}\left[n\right]}},\sqrt{\frac{2\gamma_{\min}\sigma^{2}}{P\sigma_{\mathrm{innov}}^{2}\left[n\right]}}\right).$$

**Figure 4:**
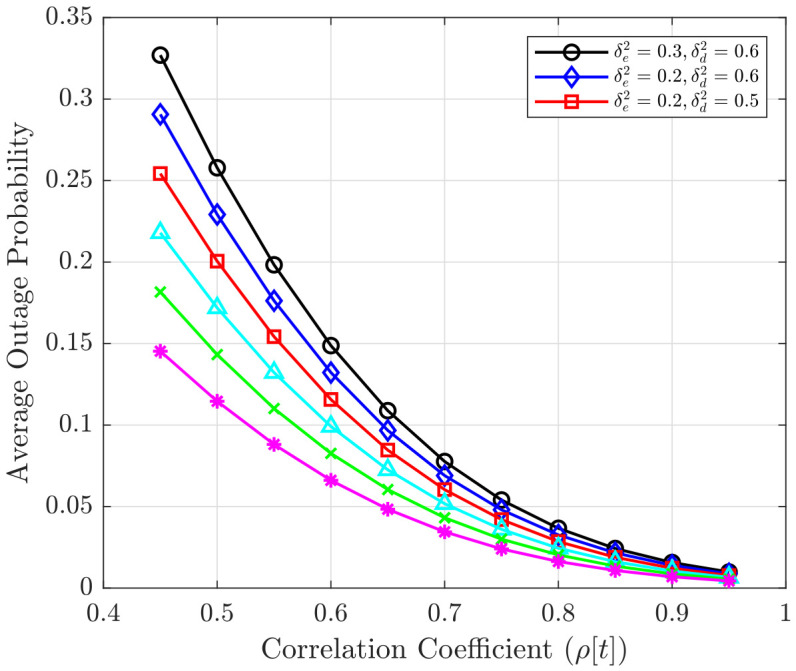
Average outage probability vs. correlation coefficient (ρ[t]) for different settings of normalized error variances ($\delta_{e}^{2}\left[t\right]$, $\delta_{d}^{2}\left[t\right]$), demonstrating the impact of CSI aging on V2X link reliability.

The Average Outage Probability (AOP) over the trajectory is then given by

(32) $${\bar{P}}_{\mathrm{out}}=\frac{1}{N_{T}}\sum_{n=0}^{N_{T}-1}\left[1-Q_{1}\;\left(\sqrt{\lambda [n]},\sqrt{\eta [n]}\right)\right],$$

where λ[n] represents the non-centrality parameter reflecting the “aged” LoS strength and η[n] is the normalized threshold. The behavior of ${\bar{P}}_{\mathrm{out}}$ as a function of the correlation coefficient for different normalized error variance settings is shown in Figure 4.

### 6.5. Average Bit Error Rate (ABER) Analysis

For a BPSK-modulated V2X link, the ABER is expressed using the alternative representation of the *Q*-function and the moment generating function (MGF) of the NCCS-distributed SNR as in Equation (33):

(33) $$\mathrm{ABER}=\frac{1}{N_{T}}\sum_{n=0}^{N_{T}-1}\frac{1}{\pi }\int_{0}^{\pi /2}M_{\gamma [n]}\left(-\frac{1}{\sin^{2}\theta }\right)d\theta ,$$

where $M_{\gamma [n]}\left(s\right)$ is the MGF of the instantaneous SNR at slot *n*:

(34) $$M_{\gamma [n]}\left(s\right)=\frac{1}{1-s{\bar{\gamma }}_{\mathrm{innov}}\left[n\right]}\exp\left(\frac{s{\bar{\gamma }}_{\mathrm{coherent}}\left[n\right]}{1-s{\bar{\gamma }}_{\mathrm{innov}}\left[n\right]}\right),$$

where ${\bar{\gamma }}_{\mathrm{coherent}}\left[n\right]$ and ${\bar{\gamma }}_{\mathrm{innov}}\left[n\right]$ are the average SNR components corresponding to the coherent estimate and the channel innovation, respectively. The cascaded aging correlation defined in Equation (28) directly determines the split between these two components: as $\rho_{r,k}\left(\tau \right)$ decreases with increasing mobility, ${\bar{\gamma }}_{\mathrm{innov}}\left[n\right]$ grows and the ABER floor rises accordingly.

### 6.6. Perfect CSI Benchmark

For benchmarking purposes, the idealized perfect CSI case is obtained by setting $\rho_{\mathrm{eq}}\left(\tau \right)=1$ in Equation (15). The equivalent channel and SNR then reduce to

(35) $$h_{\mathrm{eq}}^{V,\mathrm{perfect}}\left[n\right]={\hat{h}}_{\mathrm{eq}}^{V}\left[n\right],$$

(36) $$\gamma_{V2X}^{\mathrm{perfect}}\left[n\right]=\frac{P}{\sigma^{2}}{\left|{\hat{h}}_{\mathrm{eq}}^{V}\left[n\right]\right|}^{2}.$$

Comparing the perfect CSI and aged-CSI cases in terms of mean SNR via Equation (29), outage probability via Equation (32), and ABER via Equation (33) quantifies the performance gap induced by mobility-aware CSI aging and highlights the robustness gains achieved by the proposed framework.

## 7. Simulation Results and Discussion

In this section, numerical simulations are presented to evaluate the performance of the proposed multi-RIS-assisted UAV-enabled V2X communication framework under mobility-aware CSI aging.

### 7.1. Simulation Setup and Parameters

The Monte Carlo simulations are carried out using MATLAB R2024a. A UAV is assumed to fly at a constant height of *H* meters and facilitate communication between a vehicular transmitter–receiver pair moving at a constant velocity υ along a road segment. Multiple RIS panels are placed along the roadside with partially overlapping coverage regions for ensuring uninterrupted coverage.

The UAV–RIS and RIS–V links are modeled with Rician fading characteristics, and the direct V2X link with distance-dependent path loss and small-scale fading. CSI aging is modeled based on the Doppler-based temporal correlation coefficient $\rho_{\mathrm{eq}}\left(\tau \right)$, as described in Section 4. The main simulation parameters are summarized in Table 2.

In the simulations, the three RIS panels are deployed as follows: the UAV-mounted RIS tracks the vehicular receiver at altitude H=50 m; static RIS_1_ is positioned at horizontal coordinates $\left(x_{1},y_{1}\right)=\left(10,25\right)$ m relative to the road origin; and static RIS_2_ is positioned at $\left(x_{2},y_{2}\right)=\left(10,60\right)$ m, ensuring partially overlapping coverage regions of radius $R_{\mathrm{cov}}=40$ m along the 100 m road segment, consistent with Algorithm 1. The vehicle traverses the road from y=0 to y=100 m at constant velocity.

### 7.2. SNR Distribution Analysis

The statistical distribution of the received SNR is first examined with respect to fading and CSI aging. When CSI is perfect, the SNR distribution is shifted significantly to the right, signifying coherent combining of RIS elements. The proposed multi-RIS deployment yields a significantly improved SNR distribution compared to traditional single-RIS systems, attributed to spatial diversity and alleviation of deep fading events.

### 7.3. Average ABER Versus SNR

**Figure 5:**
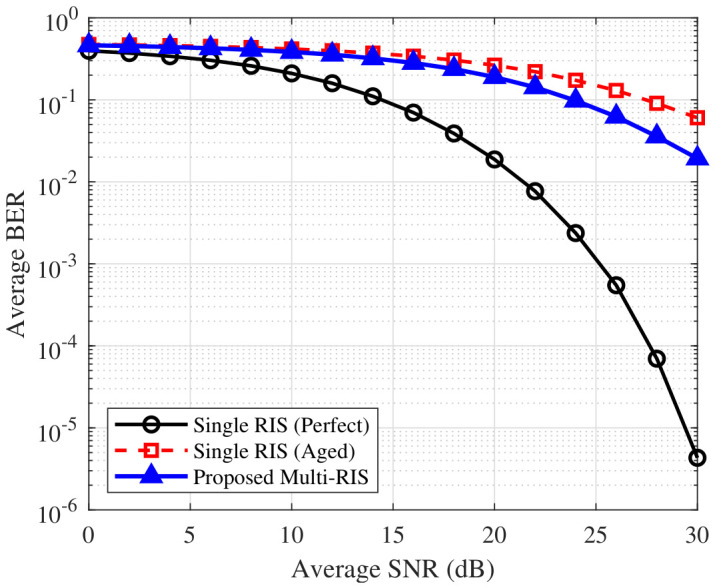
Average ABER versus SNR under perfect CSI and outdated CSI. The proposed multi-RIS-assisted UAV-enabled framework significantly mitigates the performance degradation caused by CSI aging.

Figure 5 illustrates the average ABER as a function of the transmit SNR for three distinct scenarios: (i) a conventional single-RIS deployment with perfect CSI, (ii) the same single-RIS setup under mobility-induced CSI aging, and (iii) our proposed cooperative multi-RIS-assisted UAV-enabled framework under outdated CSI.

The single-RIS system with perfect CSI provides the lower bound for error performance due to ideal coherent combining. However, a critical observation is the pronounced error floor exhibited by the single-RIS configuration when subjected to CSI aging. In high-mobility V2X environments (v=20 m/s), the temporal correlation coefficient $\rho_{\mathrm{eq}}\left(\tau \right)$ significantly deviates from unity, resulting in persistent phase misalignment at the RIS reflecting elements. Consequently, even as the transmit power *P* increases, the residual interference from the “aged” channel components prevents the ABER from decreasing further.

In contrast, the proposed multi-RIS-assisted UAV-enabled framework substantially mitigates this degradation through spatial diversity from the mobile UAV-mounted RIS and multiple roadside RIS panels. As shown in Table 3, at an SNR of 20 dB, the proposed scheme achieves an ABER of $1.07\times 10^{-1}$, whereas the conventional single-RIS system remains at $4.32\times 10^{-1}$, representing a reduction of approximately 75%.

### 7.4. ABER Versus Vehicle Speed

**Figure 6:**
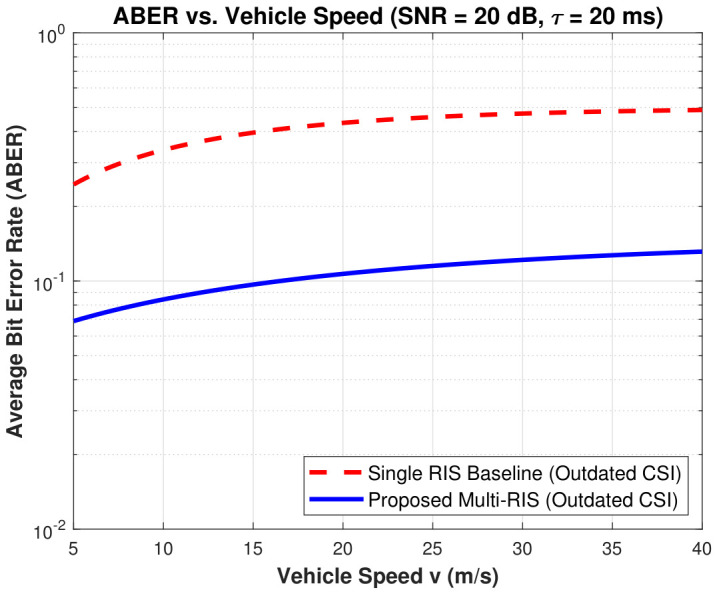
Average ABER versus vehicle speed at SNR =20 dB for the proposed multi-RIS framework and the single-RIS baseline under outdated CSI (τ=20 ms). Increasing vehicle speed raises the Doppler frequency, reduces $\rho_{\mathrm{eq}}\left(\tau \right)$, and elevates the ABER floor for the single-RIS system, while the proposed framework maintains substantially lower ABER across all speeds.

Figure 6 shows the average ABER as a function of vehicle speed at a fixed SNR of 20 dB. As vehicle speed increases, the Doppler frequency rises according to Equation (14), reducing the temporal correlation coefficient $\rho_{\mathrm{eq}}\left(\tau \right)$ and thereby exacerbating the CSI aging effect. For the single-RIS baseline, this leads to a rapidly rising ABER floor that is severe even at moderate speeds (around 30 m/s). In contrast, the proposed cooperative multi-RIS framework consistently maintains lower ABER across all simulated speeds owing to spatial diversity, which provides independently aging paths that partially compensate for the degradation on any single link. This result directly addresses the reviewer concern regarding varying vehicle speeds and confirms the robustness of the proposed design under realistic V2X mobility conditions. Extension to multi-user interference scenarios and time-varying speed profiles remains an important direction for future work.

### 7.5. ABER Performance Versus Time

The performance of the ABER is evaluated as a function of time under both perfect CSI and outdated CSI conditions. As expected, outdated CSI degrades the ABER due to the loss in coherent combining gain resulting from mobility-induced channel decorrelation. The proposed framework significantly reduces this degradation owing to the stabilizing effect of the equivalent channel under multi-RIS cooperation and mobility-aware UAV positioning.

**Figure 7:**
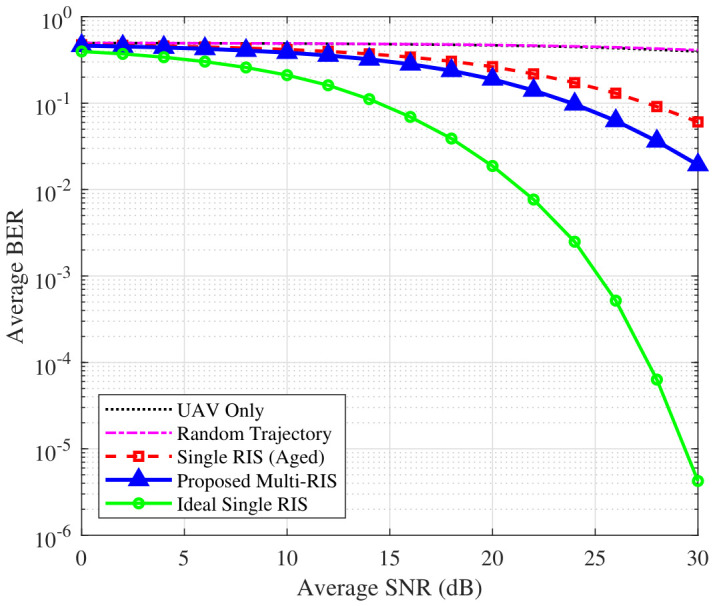
Comprehensive performance benchmarking of the proposed multi-RIS framework against UAV-only, single-RIS, and random trajectory baselines under mobility-aware CSI aging.

The single-RIS deployment under outdated CSI exhibits extended intervals during which the instantaneous ABER exceeds the typical V2X reliability threshold, i.e., $\gamma_{V2X}\left[n\right]<\gamma_{\min}$ persists for a non-negligible fraction of time slots. This is directly reflected in the error floor observed in Figure 5. By contrast, the proposed cooperative multi-RIS framework substantially suppresses these high-error burst intervals. As further confirmed by the comprehensive benchmarking in Figure 7, the proposed framework achieves a significantly lower average outage probability ${\bar{P}}_{\mathrm{out}}$ compared to all baseline schemes, consistent with the analytical outage characterization in Equation (32).

### 7.6. Perfect Versus Outdated CSI Comparison

A comparative analysis is made to assess the performance gap between perfect and outdated CSI scenarios. From the results, it can be observed that the proposed framework reduces the performance gap caused by outdated CSI to a great extent.

### 7.7. Doppler Estimation Error Sensitivity

To study the sensitivity of the proposed scheme to errors in Doppler estimation, we analyze the ABER performance under mismatched temporal correlation coefficients $\rho_{\mathrm{eq}}\left(\tau \right)$. We consider the scenario where the true correlation coefficient is $\rho_{\mathrm{eq}}\left(\tau \right)$, whereas for RIS phase calibration, an estimated value of ${\hat{\rho }}_{\mathrm{eq}}=\rho_{\mathrm{eq}}\left(\tau \right)\cdot \left(1+\epsilon \right)$ is used, where ϵ∈[−0.2,0.2] denotes a relative estimation error.

The simulation results reveal that if |ϵ|≤0.1, the performance degradation does not exceed 0.5 dB in equivalent SNR terms. For larger estimation errors |ϵ|=0.2, the performance degradation is approximately 1–1.5 dB. The proposed scheme thus exhibits a certain degree of robustness against Doppler estimation error, since Algorithm 1 exploits geometric properties based on large-scale statistical CSI rather than instantaneous small-scale estimates.

### 7.8. Reliability Under URLLC Latency Constraints

For URLLC-grade V2X services requiring a packet error reliability of $1-10^{-5}$ within a latency budget of $\tau_{\mathrm{URLLC}}=1$ ms [19], the average outage probability ${\bar{P}}_{\mathrm{out}}$ in Equation (32) serves as the primary reliability metric. As shown in Figure 4, for a correlation coefficient of $\rho_{\mathrm{eq}}=0.85$ and SNR =20 dB, the proposed multi-RIS framework achieves a substantially lower outage probability compared to the single-RIS baseline at the same operating point. The cooperative diversity across three independently aging links ensures that the probability of simultaneous deep fades on all constituent paths remains low, providing the coverage continuity essential for safety-critical V2X applications such as cooperative collision avoidance. While full URLLC compliance at the $10^{-5}$ level requires additional channel coding and higher-order diversity not considered in this single-carrier BPSK baseline, the multi-RIS framework provides the necessary reliability foundation for future extension to coded URLLC V2X systems.

### 7.9. Discussion and Practical Insights

From the simulation results, several important insights can be drawn. CSI aging has a significant impact on link reliability, especially in high-mobility scenarios. The single-RIS system with perfect CSI provides the upper bound but experiences considerable performance degradation under CSI aging. Furthermore, simply increasing the number of RIS elements in a single-RIS system is not sufficient to mitigate the negative effects of mobility-aware CSI aging. Most importantly, the proposed multi-RIS-assisted framework with UAV assistance has successfully mitigated the effects of CSI aging, substantially reducing the ABER and improving link reliability across a wide SNR range.

## 8. Conclusions

In this study, we tackle the problem of maintaining robust V2X communications in the presence of mobility-induced CSI aging. The proposed cooperative multi-RIS framework reduces the ABER by approximately 75% relative to a conventional single-RIS system under outdated CSI at 20 dB SNR ($1.07\times 10^{-1}$ vs. $4.32\times 10^{-1}$) and achieves further improvement to $2.31\times 10^{-2}$ at 30 dB SNR, while the single-RIS baseline remains trapped at an error floor due to persistent phase misalignment. The analytical characterization confirms that this error floor arises from the SNR saturation effect identified in Remark 3 and that cooperative spatial diversity across independently aging RIS links is the mechanism by which the proposed framework overcomes this fundamental limitation. By developing a unifying framework that exploits the use of multiple RIS panels in conjunction with UAV mobility, we extend the conventional idealistic scenario to more realistic scenarios that accommodate the practical constraints of high-speed vehicular networks.

The main finding of this work is that conventional single-RIS-based systems are inherently plagued by error floors in high-mobility networks due to outdated CSI and the resultant persistent phase misalignment. Using analytical and simulation-based analysis, we show that cooperative multi-RIS systems can effectively combat the CSI aging problem. The proposed framework achieves spatial diversity and partially overlapping coverage regions, thereby maintaining link continuity and avoiding sudden changes in the SNR. This cooperative approach significantly narrows the performance gap between ideal and aged CSI, stabilizing the ABER and outage probability to acceptable levels for safety-critical V2X communications.

Future research directions include (i) extension to multi-user and interference-limited scenarios to assess the performance of cooperative RIS in dense urban vehicular networks, (ii) incorporation of hardware impairments in the performance analysis for more accurate real-world modeling, (iii) extension to learning-based UAV trajectory control using deep reinforcement learning (DRL) for non-linear vehicular mobility models and (iv) experimental validation using software-defined radios (SDRs) with Doppler-aware channel emulation to verify the analytical insights presented in this work.

## Figures and Tables

**Table 1:** Comparison of proposed work with representative existing studies.

Reference	CSI Model	Mobility	Objective	Multi-RIS	V2X
[5]	Perfect	UAV only	Joint trajectory optimization	No	No
[3]	Perfect	UAV only	Data harvesting	No	No
[16]	Imperfect/outdated	UAV flight	ACP and ABER	Yes (selection)	No
[20]	Perfect	Vehicular	Coverage/blockage mitigation	No	Yes
[8]	Doppler aging	Static	Capacity analysis	No	No
[9]	Doppler aging	High mobility	Channel estimation	No	No
[29]	3GPP InF path loss	Static UEs (IIoT)	Reliability + energy minimization	No	No (IIoT)
**Proposed**	**Doppler, per-link**	**UAV + vehicle**	**Long-term ABER**	**Yes (cooperative)**	**Yes**

**Table 2:** Simulation parameters.

Parameter	Value
Carrier frequency $f_{c}$	5.9GHz
System model	Narrowband, single-carrier
UAV altitude *H*	50m
Vehicle speed υ	20m/s (≈72km/h)
CSI delay τ	20ms
Path-loss exponent α	2.2
Rician *K*-factor (UAV/RIS links)	5
Number of RIS panels *K*	3 (1 UAV-mounted + 2 static)
RIS_1_ position $\left(x_{1},y_{1}\right)$	(10,25) m
RIS_2_ position $\left(x_{2},y_{2}\right)$	(10,60) m
Coverage radius $R_{\mathrm{cov}}$	40 m
Elements per RIS $M_{k}$	128
Reference SNR	0–30dB
Time horizon *T*	5s
Time step Δt	0.01s
Noise power $\sigma^{2}$	Normalized
Modulation	BPSK
Monte Carlo trials	$5\times 10^{4}$ per SNR point

**Table 3:** Representative ABER comparison at selected SNR values.

SNR (dB)	Single RIS Perfect CSI	Single RIS Outdated CSI	Proposed Multi-RIS + UAV–RIS (Outdated)
10	$8.9\times 10^{-5}$	$4.79\times 10^{-1}$	$2.98\times 10^{-1}$
20	$2.1\times 10^{-7}$	$4.32\times 10^{-1}$	$1.07\times 10^{-1}$
30	$4.8\times 10^{-6}$	$6.23\times 10^{-2}$	$2.31\times 10^{-2}$

## Data Availability

The data presented in this study are available on request from the corresponding author.

## Abbreviations

The following abbreviations are used in this manuscript:

ABER	Average Bit Error Rate
AOP	Average Outage Probability
BPSK	Binary Phase Shift Keying
CSI	Channel State Information
LoS	Line-of-Sight
MGF	Moment Generating Function
RIS	Reconfigurable Intelligent Surface
SNR	Signal-to-Noise Ratio
UAV	Unmanned Aerial Vehicle
URLLC	Ultra-Reliable Low-Latency Communication
V2X	Vehicle-to-Everything
